# Comparison of Design, Eligibility, and Outcomes of Neuroendocrine Neoplasm Trials Initiated From 2000 to 2009 vs 2010 to 2020

**DOI:** 10.1001/jamanetworkopen.2021.31744

**Published:** 2021-10-27

**Authors:** Satya Das, Liping Du, Cody L. Lee, Nina D. Arhin, Jennifer A. Chan, Elise C. Kohn, Daniel M. Halperin, Jordan Berlin, Heather LaFerriere, Simron Singh, Pamela L. Kunz, Arvind Dasari

**Affiliations:** 1 Division of Hematology and Oncology, Department of Medicine, Vanderbilt University Medical Center, Nashville, Tennessee; 2 Department of Biostatistics, Vanderbilt University Medical Center, Nashville, Tennessee; 3 Dana Faber Cancer Center, Boston, Massachusetts; 4 National Cancer Institute, Bethesda, Maryland; 5 Division of Cancer Medicine, Department of Gastrointestinal Medical Oncology, The University of Texas MD Anderson Cancer Center, Houston; 6 Biomedical Library, Vanderbilt University, Nashville, Tennessee; 7 Sunybrook Health Sciences Centre, Toronto, Canada; 8 Yale Cancer Center, New Haven, Connecticut

## Abstract

**Importance:**

Neuroendocrine neoplasms (NENs) have historically been grouped homogenously in clinical trials, despite their heterogeneity. Given the adoption of a more advanced pathologic classification system and drug licensure of several targeted therapies over the last decade, information is needed on whether study characteristics of NEN studies have evolved.

**Objective:**

To assess changes in study design, eligibility, accrual, sponsorship, and outcomes between phase II or III NEN clinical trials that began enrollment from 2000 to 2009 vs 2010 to 2020.

**Design, Setting, and Participants:**

This quality improvement study used a systematic survey of completed studies published between January 1, 2000, and December 31, 2020. Therapeutic phase II and III NEN studies were identified through a database search of Medline (via PubMed), EMBASE (OvidSP), Cumulative Index of Nursing and Allied Health Literature (EBSCOhost), Web of Science (Clarivate), Cochrane Database of Systematic Reviews (Wiley), ClinicalTrials.gov (National Institutes of Health), EU Clinical Trials Register, and National Cancer Institute Clinical Trials. Data were analyzed between March and June 2021.

**Main Outcomes and Measures:**

Study characteristic proportions between the 2 enrollment periods.

**Results:**

Of 3243 identified studies, 119 studies met criteria for inclusion, of which 117 studies (54 studies that began enrollment between 2000-2009 and 63 studies that began enrollment between 2010-2020) included exact dates of enrollment and were compared. Studies that began enrollment after 2010, compared with studies that began enrollment from 2000 to 2009, were less likely to include all NENs (13 studies [21%] vs 34 studies [63%]; *P* < .001) and more likely to include select NENs (eg, gastrointestinal neuroendocrine tumors, 25 studies [40%] vs 11 studies [20%]; *P* = .02; pancreatic neuroendocrine tumors, 32 studies [51%] vs 16 studies [30%]; *P* = .02). Studies that began enrollment after 2010, compared with studies that began enrollment from 2000 to 2009, were more likely to specify tumor differentiation (59 studies [98%] vs 34 studies [63%]; *P* < .001) or Ki-67 index (23 studies [38%] vs 5 studies [9%]; *P* < .001) in inclusion criteria. Studies that began enrollment after 2010, compared with studies that began enrollment from 2000 to 2009, were more likely to use progression-free survival (22 studies [35%] vs 9 studies [18%]; *P* = .04) rather than objective response rate (19 studies [30%] vs 27 studies [53%]; *P* = .01) as a primary or coprimary end point.

**Conclusions and Relevance:**

These findings suggest that NEN trials enrolling over the last decade were more focused on select tumor populations, compared with studies that began enrollment before 2010. Despite this shift, more than 20% of studies still included all NENs. Studying novel agents in specific disease populations may enhance drug development in the field.

## Key Points

**Question:**

Have study characteristics of studies in patients with neuroendocrine neoplasms (NENs) changed over the last 2 decades (2000-2009 vs 2010-2020)?

**Findings:**

In this quality improvement study, 119 therapeutic phase II and III studies were identified after conducting a systematic survey of NEN studies completed between January 2000 and December 2020. Studies that began enrollment after 2010 were less likely to include all NENs and more likely to specify tumor differentiation or Ki-67 index in inclusion criteria compared with studies that began enrollment before 2010.

**Meaning:**

These findings suggest that NEN studies enrolling over the last decade became more focused on select tumor populations and specifically distinguishing neuroendocrine tumors from neuroendocrine carcinomas.

## Teaser

This quality improvement study assesses changes in study design, eligibility accrual, sponsorship, and outcomes between neuroendocrine neoplasm studies that began enrollment from 2000 to 2009 vs 2010 to 2020.

## Introduction

Neuroendocrine neoplasms (NENs) are heterogenous tumors with incidence and prevalence that continue to increase steadily.^1^ Despite their heterogeneity, NENs have historically been grouped homogenously in clinical trials. A prior publication examining the design of therapeutic phase II and III clinical trials in patients with gastroenteropancreatic neuroendocrine tumors (NETs) identified limitations in these studies, such as the inclusion of multiple tumor types, tumors with unspecified differentiation or grade, and nonprogressive tumors.^2^ Over the last decade, the adoption of a more advanced pathological classification system has significantly influenced NEN categorization. In particular, the most pronounced addition to the 2019 World Health Organization pathologic classification system for NENs was the definition of the grade 3 well-differentiated NET category, which furthered the distinction between well-differentiated NETs and poorly differentiated neuroendocrine carcinomas (NECs).^3,4^ Also over this period of time, from 2010 to 2020, several targeted therapies (ie, everolimus and sunitinib) were licensed by national regulatory bodies and clinical trial design recommendations from the 2011 National Cancer Institute (NCI) NET Clinical Trials Planning Meeting were disseminated.^5,6,7,8^ Given these advances, in this analysis, we sought to assess whether there had been changes in study design, eligibility, accrual, sponsorship, and outcomes between phase II or III NEN studies that began enrollment between 2000 and 2009 vs between 2010 and 2020.

## Methods

### Search Strategy

This quality improvement study was deemed exempt from review by the institutional review board of Vanderbilt University Medical Center because no identifiable patient data were accessed. This study follows the Standards for Quality Improvement Reporting Excellence (SQUIRE) reporting guideline. A literature search was performed to identify all phase II and phase III therapeutic studies in patients with NENs published (including abstracts) between January 1, 2000, and December 31, 2020. Medline (via PubMed), EMBASE (OvidSP), Cumulative Index of Nursing and Allied Health Literature (EBSCOhost), Web of Science (Clarivate), Cochrane Database of Systematic Reviews (Wiley), ClinicalTrials.gov (National Institutes of Health), European Union Clinical Trials Register, and NCI Clinical Trials were searched in December 2020 for relevant studies.

The search strategy was a combination of keywords and database-specific subject headings, including the following search terms and variations of each term: *neuroendocrine tumors*, *neuroendocrine malignancy*, *neuroendocrine carcinoma*, *neuroendocrine cancer*, *neuroendocrine neoplasms*, *clinical trials*, *randomized trials*, *controlled trials*, *drug trials*, *phase II*, and *phase III*. Trials specifically focusing on patients with small-cell lung cancer, paraganglioma, pheochromocytoma, neuroblastoma, medullary thyroid cancer, or adrenal cortical cancer were excluded and categorized as not topical. Review articles were excluded and categorized as not topical.

### Data Abstraction

A data abstraction spreadsheet was generated by 2 of us (S.D. and A.D.). Two of us (C.L. and N.A.) piloted the data abstraction tool on the initial 40 studies. A third author (S.D.) reviewed these data points to assess for concordance between abstraction techniques; interobserver agreement was 99% (1740 of 1760 points) for all data points. Thereafter, the remaining studies were abstracted by either C.L. or N.A. S.D. reviewed all study abstraction data, and any data disagreements were resolved after consensus was achieved through discussions with the abstracting author. Details of data abstraction are described in the eMethods the Supplement.

### Statistical Analysis

Frequencies and relative frequencies were used to describe the categorical variables. Proportions between the 2 enrollment periods were compared using Pearson χ^2^ tests. Continuous variables were compared between periods using Wilcoxon rank sum tests. Analyses were conducted using R statistical software version 4.0.2 (R Project for Statistical Computing). *P* values were 1-sided, and statistical significance was set at *P* = .05. Data were analyzed between March and June 2021.

## Results

Of 3243 identified studies, 119 met criteria for inclusion (eFigure 1 in the Supplement) while 117 included exact dates of enrollment, including 54 studies that began enrollment between 2000 and 2009 and 63 studies that began enrollment between 2010 and 2020. Details on included studies are summarized in the Table.

**Table.** Comparison of Study Characteristics (Enrollment Start 2000-2009 vs 2010-2020)

Category	Enrollment period, No. (%)		Total (N = 117)^a^	*P* value^b^
	2000-2009 (n = 54)	2010-2020 (n = 63)		
**Study design**				
Phase				
II	45 (83)	54 (86)	99 (85)	.72
III	9 (17)	9 (14)	18 (15)	
Design^c^				
Randomized	12 (22)	20 (32)	32 (27)	.51
Single arm	34 (63)	35 (56)	69 (59)	
Parallel cohorts, non-randomized	8 (15)	8 (13)	16 (14)	
Primary or coprimary end point^d^				
Progression-free survival	9 (18)	22 (35)	31 (27)	.04
Objective response rate	27 (53)	19 (30)	46 (40)	.01
Overall survival	1 (2)	1 (2)	2 (2)	.88
Biomarker-enriched population	10 (19)	10 (16)	20 (17)	.71
Placebo-controlled	4 (7)	8 (13)	12 (10)	.35
Single agent	31 (57)	38 (60)	69 (59)	.75
Novel agent(s)	22 (41)	22 (35)	44 (38)	.52
Agent(s) licensed by a national regulatory body	2 (4)	12 (19)	14 (12)	.01
Agent class^e^				
Anti-VEGF	15 (28)	19 (30)	34 (29)	.78
Radioligand therapy	9 (17)	6 (10)	15 (13)	.25
Immune checkpoint inhibitor	0	5 (8)	5 (4)	.03
Chemotherapy	14 (26)	12 (19)	26 (22)	.37
Non-VEGF receptor pathway inhibitor	11 (20)	22 (35)	33 (28)	.08
Cell cycle inhibitor	0	1(2)	1(1)	.35
Somatostatin analog	15 (28)	16 (25)	31 (26)	.77
Other agents	9 (16.6)	6 (9.5)	15 (12.8)	.25
Defined primary end point	51 (94)	63 (100)	114 (97)	.06
Prespecified sample size	40 (74)	46 (74)	86 (74)	.99
**Eligibility**				
NEN types specified in inclusion criteria				
All NENs^f^	34 (63)	13 (21)	47 (40)	<.001
Gastrointestinal NETs^g^	11 (20)	25 (40)	36 (31)	.02
Limited to gastrointestinal NETs	2 (4)	2 (3)	4 (3)	.88
Pancreatic NETs^g^	16 (30)	32 (51)	48 (41)	.02
Limited to pancreatic NETs	7 (13)	16 (25)	23 (20)	.09
Lung NETs^g^	4 (7)	11 (17)	15 (13)	.11
Limited to lung NETs	1 (2)	1 (2)	2 (2)	.91
NECs^g^	1 (2)	11 (17)	12 (10)	.006
Limited to NECs	0	7 (11)	7 (6)	.01
Treatment line				
First	10 (19)	8 (13)	18 (16)	.32
Later	27 (50)	39 (64)	66 (57)	
Any	17 (31)	14 (23)	31 (27)	
Disease progression required at baseline (among later line studies)	18 (42)	35 (67)	53 (56)	.01
Tumor differentiation reported in inclusion criteria^h^	34 (63)	59 (98)	93 (82)	<.001
Ki-67 Index reported in inclusion criteria^i^	5 (9)	23 (38)	28 (25)	<.001
Reported inclusion criteria	53 (98)	60 (98)	113 (98)	.93
**Accrual and sponsorship**				
Sponsorship^c^				
Industry only	24 (47)	26 (43)	50 (45)	.93
National Cancer Institute only	10 (20)	13 (22)	23 (21)	
Other only	9 (18)	13 (22)	22 (20)	
Multiple	8 (16)	8 (13)	16 (14)	
Any industry funding	30 (59)	32 (53)	62 (56)	.56
Study location				
United States only	24 (44)	17 (27)	41 (35)	.26
North America	1 (2)	3 (5)	4 (3)	
Outside United States only	20 (37)	29 (47)	49 (42)	
Global	9 (17)	13 (21)	22 (20)	
Multicenter	29 (60)	41 (72)	70 (67)	.21
Slow accrual	0	3 (5)	3 (3)	.11
Premature termination	9 (17)	14 (23)	23 (20)	.40
Reason for termination				
Poor accrual^j^	1 (11)	2 (14)	3 (13)	.83
Other^k^	8 (89)	12 (86)	20 (87)	
Sample size discrepancy^l^	10 (24)	16 (35)	26 (30)	.29
**Outcomes**				
Regulatory licensing for an agent	3 (6)	5 (8)	8 (7)	.61
Positive primary end point	33 (65)	37 (66)	70 (65)	.88
Objective response rate ≥10%	25 (50)	26 (57)	51 (53)	.52
Results reported as a published manuscript	50 (93)	42 (67)	92 (79)	<.001

Abbreviations: NEC, neuroendocrine carcinoma; NEN, neuroendocrine neoplasm; NET, neuroendocrine tumor; VEGF, vascular endothelial growth factor.

^a^ Only 117 studies were included because 2 studies did not specify year of enrollment start.

^b^ Calculated using Pearson χ^2^ test.

^c^ Owing to rounding, the categories may add up to more than 100%.

^d^ Not mutually exclusive: if a study included coprimary end points, each primary end point was counted.

^e^ Not mutually exclusive: if a study included agents of different classes, each agent class was counted.

^f^ Studies that enrolled all NENs did not specify primary tumor site or tumor differentiation in inclusion criteria.

^g^ Not mutually exclusive: if a study specified the inclusion of several tumor types, each tumor type was counted.

^h^ Tumor differentiation was documented as being defined in study eligibility criteria if well-differentiated or poorly differentiated histological findings were explicitly stated. Low-, intermediate-, or high-grade was also accepted as a surrogate for this measure.

^i^ Ki-67 index was documented as being defined in study eligibility criteria if Ki-67 percentages were explicitly listed. Grade listings (grade 1, 2, or 3) were also accepted as a surrogate for this measure.

^j^ Poor accrual differs from slow accrual. A slow accruing study was defined as one that enrolled fewer than 2 patients per year. Poor accrual was used as a justification for terminating a study without a formal definition.

^k^ Of these studies, 15 were closed owing to lack of efficacy in an interim analysis while 5 were closed owing to meeting the efficacy threshold in an interim analysis.

^l^ Sample size discrepancy was denoted if the actual study sample size was at least 10 patients fewer than the predefined study sample size.

### Study Design and Eligibility

Studies that began enrollment after 2010, compared with studies that began enrollment between 2000 and 2009, were less likely to include all NENs (13 studies [21%] vs 34 studies [63%]; *P* < .001) and were more likely to specify site of origin (eg, gastrointestinal NETs, 25 studies [40%] vs 11 studies [20%]; *P* = .02; pancreatic NETs, 32 studies [51%] vs 16 studies [30%]; *P* = .02) or grade (NECs, 11 studies [17%] vs 1 study [2%]; *P* = .006). Studies beginning enrollment in 2010, compared with studies beginning enrollment before 2010, were more likely to specify tumor differentiation (59 studies [98%] vs 34 studies [63%]; *P* < .001), Ki-67 index (23 studies [38%] vs 4 studies [9%]; *P* < .001), and, among later-line studies, mandated disease progression at study entry (35 studies [67%] vs 18 studies [42%]; *P* = .01) in inclusion criteria.

Studies that began enrollment after 2010 were more likely to use progression-free survival (PFS) (35% vs 18%, *P* = .04) over objective response rate (ORR) (19 studies [30%] vs 27 studies [53%]; *P* = .01) as a primary or coprimary end point compared with studies that began enrollment before 2010.

**Figure 1.**
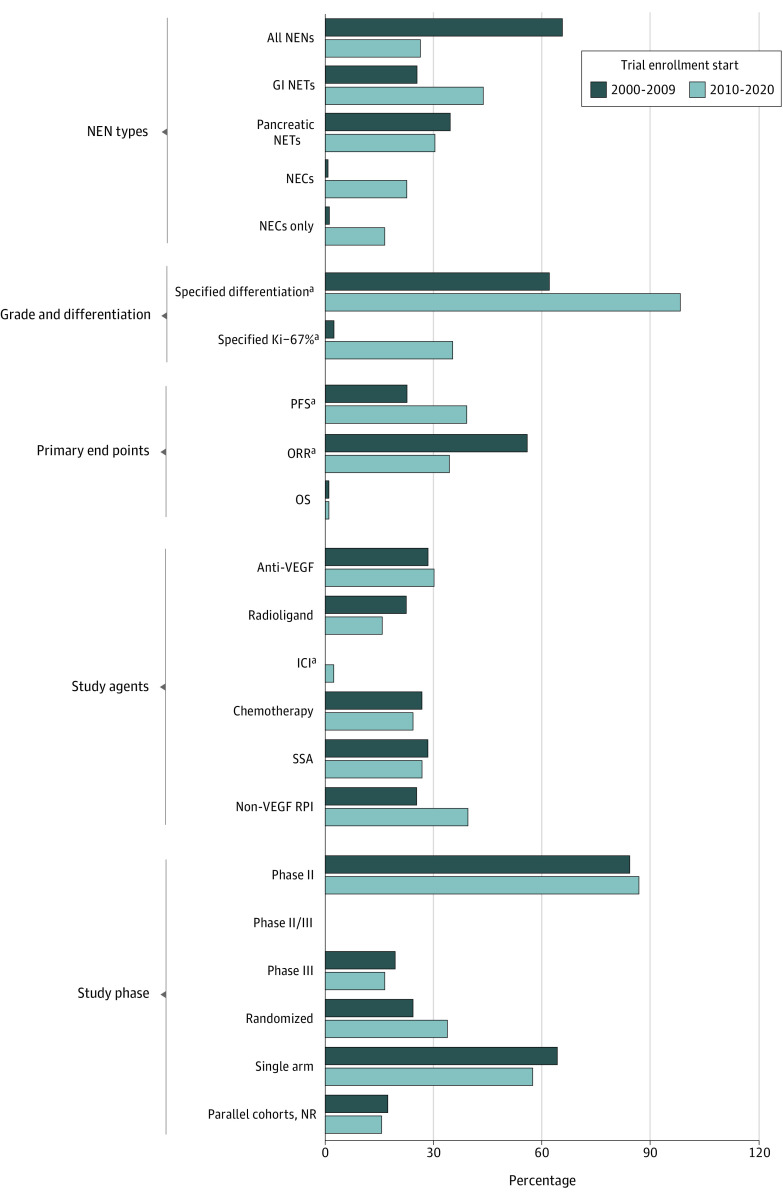
Comparison of Study Design and Eligibility Characteristics of Trials That Began Enrollment Between 2000 and 2009 or Between 2010 and 2020 GI indicates gastrointestinal; ICI, immune checkpoint inhibitor; NECs, neuroendocrine carcinomas; NEN, neuroendocrine neoplasm; NETs, neuroendocrine tumors, NR, non-randomized; ORR, objective response rate; OS, overall survival; PFS, progression-free survival; RPI, receptor pathway inhibitor; SSA, somatostatin analog; and VEGF, vascular endothelial growth factor. ^a^Statistically significant difference between the enrollment periods.

A larger proportion of studies in the later enrollment period used an agent licensed by a national regulatory body (12 studies [19%] vs 2 studies [4%]; *P* = .01), whereas no change was observed in the proportion of studies using a novel agent (22 studies [35%] vs 22 studies [41%]; *P* = .52). Immune checkpoint inhibitors were the only agent class used more in studies that began enrollment after 2010 compared with those that began enrollment before (5 studies [8%] vs 0 studies; *P* = .03). Figure 1 compares key design and eligibility characteristics of studies between the enrollment periods.

### Study Accrual, Sponsorship, and Outcomes

**Figure 2.**
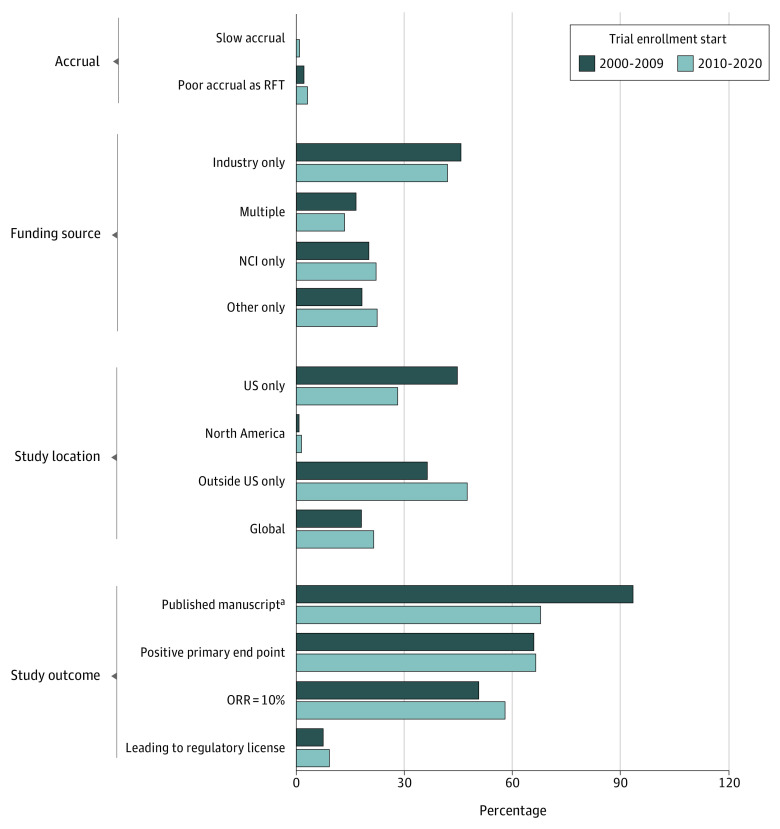
Comparison of Study Enrollment, Sponsorship, and Outcome Characteristics of Trials That Began Enrollment Between 2000 and 2009 or Between 2010 and 2020 NCI indicates National Cancer Institute; ORR, objective response rate . ^a^Statistically significant difference between the enrollment periods.

The median (range) accrual rates were 1.53 (0.15-13.3) patients per month in the phase II studies and 5.08 (0.03-25.16) patients per month in phase III studies, with no difference in median accrual rates between enrollment periods (median [IQR], 1.65 [0.79-3.83] patients per month in later period studies vs 1.78 [1.20-3.35] patients per month in earlier period studies; *P* = .36). The median (range) sample sizes were 37 (8-171) patients in phase II studies and 142.5 (1-410) patients in phase III studies, with no difference in median sample sizes between periods (median [IQR], 37 [22-77] patients in later period studies vs 43 [27-65] patients in earlier period studies; *P* = .90). Study location and sponsorship distributions did not differ between study periods (Table). There were no differences in the proportion of studies that met their primary end point nor in the proportion of studies in which the experimental agent achieved an objective response rate of 10% or greater. Figure 2 compares key accrual and outcome characteristics of studies between the enrollment periods. Fourth, among 23 terminated studies, only 3 studies (13%) were owing to poor accrual.

### Exploratory Analysis 

To assess the continuing evolution of NEN studies, we compared study characteristics between currently enrolling studies and the completed studies in our analysis (eFigure 2 in the Supplement). Although the number of currently enrolling studies was limited, statistically significant increases in the proportion of studies enrolling NECs only (8 studies [33%] vs 7 studies [6%]; *P* < .001) and using radioligand therapy (10 studies [42%] vs 15 studies [13%]; *P* = .001) or chemotherapy (12 studies [50%] vs 27 studies [23%]; *P* = .006) were observed; a statistically significant decrease in the proportion of RCTs using placebo control (1 study [6%] vs 12 studies [38%]; *P* < .001) was also observed.

## Discussion

In this quality improvement study, we describe the study characteristics of phase II and III NEN studies published between 2000 and 2020 and compare characteristics of studies that began enrollment before or after 2010. Several insights were noted. First, a greater proportion of studies that began enrollment after 2010 included only NECs and specified tumor differentiation or Ki-67 index in eligibility criteria. These trends reflect efforts to distinguish NETs from NECs in studies, given the vast differences in tumor biological characteristics, prognosis, and treatments between the tumor types, along with the adoption of an updated pathologic classification system for NENs.^3,4,9,10^ Second, a greater proportion of studies that began enrollment after 2010 included select rather than general NEN populations. This trend signals the acknowledgment by researchers in the field that NENs, based on primary tumor origin or grade, demonstrate differing sensitivities to differing therapeutics.^9^ Third, an increased proportion of studies enrolling after 2010 used PFS compared to objective response rate as a primary or coprimary end point. In contrast to many tumor types, PFS correlates with overall survival in NETs and appears to be an acceptable surrogate end point^11^; objective response rate is not uniformly associated with overall survival in NETs and has not always been measured consistently across studies (RECIST vs Southwestern Oncology Group criteria for assessing tumor response vs World Health Organization Response Evaluation Criteria in Solid Tumors).^12,13^ Fourth, among terminated studies, only 13% were owing to poor accrual. This rate compares favorably with documented rates of poor accrual leading to study termination in broader cancer populations,^14^ suggesting that despite the relative rarity of NENs, studies in this patient population have accrued successfully. Fifth, although industry was the predominant individual sponsor for studies, the proportion of studies using industry funding did not differ between enrollment periods. This finding may be owing to the distribution of studies included in the analysis. In contrast to a recent publication that highlighted the increase in industry sponsorship for cancer RCTs over the last decade,^15^ most studies included in our analysis were nonrandomized.

Finally, to assess the continuing evolution of NEN studies, we compared study characteristics between currently enrolling studies and the completed studies in our analysis, and we found statistically significant increases in the proportion of studies enrolling NECs only and using radioligand therapy or chemotherapy and a statistically significant decrease in the proportion of RCTs using placebo control. The former trends reflect efforts to build on the prior success of radioligand therapy in NETs and establish new treatment standards, centered on chemotherapy, in NECs. The latter trend points toward the increased number of licensed therapeutics for NENs and, concomitantly, an increased number of active controls to compare against experimental agents in RCTs.

### Limitations

This study has some limitations. One important limitation of our study is the possible underrepresentation of studies that began enrollment between 2000 and 2010. Study registration requirements to national trial databases were less rigorous during this initial enrollment period, and it is possible fewer studies were identified. Another limitation of this study is that the data abstraction was performed by 2 different individuals, creating the possibility of heterogenous data abstraction. Interobserver agreement was 99% for all data points from the initial 40 studies that were abstracted by both authors, making this less likely.

## Conclusions

This quality improvement study found that NEN studies enrolling during the last decade have become more focused on select tumor populations. Despite this shift, 21% of studies still included all NENs. Studying novel agents in specific disease populations may enhance drug development in the field.

## Notes

Supplement.
eMethods.
**eFigure 1.** PRISMA Diagram Depicting How the Studies Included in the Analysis Were Chosen **eFigure 2.** Comparison of Study Characteristics Between Currently Enrolling Trials and Completed Studies Included in Our Analysis
