# Supplementary material for: Comparison of Design, Eligibility, and Outcomes of Neuroendocrine Neoplasm Trials Initiated From 2000 to 2009 vs 2010 to 2020
Source: JAMA Netw Open. 2021 Oct 27;4(10):e2131744. doi: 10.1001/jamanetworkopen.2021.31744 (PMC8552059; doi:10.1001/jamanetworkopen.2021.31744)
Supplement: Supplement. — eMethods. eFigure 1. PRISMA Diagram Depicting How the Studies Included in the Analysis Were Chosen eFigure 2. Comparison of Study Characteristics Between Currently Enrolling Trials and Completed Studies Included in Our Analysis [file jamanetwopen-e2131744-s001.pdf]

Supplemental Online Content

Das S, Du L, Lee CL, et al. Comparison of design, eligibility, and outcomes of neuroendocrine neoplasm trials initiated from 2000 to 2009 vs 2010 to 2020. *JAMA Netw Open*. 2021;4(10):e2131744. doi:10.1001/jamanetworkopen.2021.31744

eMethods.

eFigure 1. PRISMA Diagram Depicting How the Studies Included in the Analysis Were Chosen

eFigure 2. Comparison of Study Characteristics Between Currently Enrolling Trials and Completed Studies Included in Our Analysis

This supplemental material has been provided by the authors to give readers additional information about their work.

© 2021 Das S et al. *JAMA Network Open.*

eMethods

Data Abstraction Details

Clinicaltrials.gov or EudraCT were used to abstract most information about specific study characteristics. In cases where discrepancies between clinicaltrials.gov or EudraCT and study publications were noted, the data from the study publication was used. Discrepancies between the clinical trial sites and study publications were noted in 8 instances (2 cases where goal sample size listing differed, 1 case where actual sample size listing differed, 3 instances where the number of enrolling centers listed varied and 2 instances where the listed starting date of enrollment differed).

Studies which enrolled all neuroendocrine neoplasms (NENs) did not specify primary tumor site or tumor differentiation in inclusion criteria. Sponsorship for cooperative group studies was attributed to national cancer institutes unless otherwise stated. The other category for funding source included institutions or foundations. Phase I/II studies were considered as phase II studies so long as only neuroendocrine tumor (NET) patients were enrolled, however only the phase II endpoints were recorded. An agent was defined as novel if it possessed a unique mechanism of action which was not emulated by a prior drug tested in NET patients. With regards to vascular endothelial growth factor (VEGF) inhibitors, if an agent targeted different VEGF receptors or other receptor pathways beyond VEGF, the agent was considered novel (e.g. lenvatinib compared to sunitinib). With regards to chemotherapy, if an agent created cell death or damage through a different mechanism compared to a prior chemotherapy, it was considered novel (e.g. pemetrexed compared to topotecan). An objective response rate (ORR) of ≥ 10% was considered clinically meaningful because of the dearth of existing cytoreductive treatments for patients with

© 2021 Das S et al. *JAMA Network Open.*

NENs. Only two therapies (^{177}Lu-Dotatate and capecitabine plus temozolomide) have demonstrated an ORR of ≥ 10% in randomized studies.

A slow accruing study was defined as one which enrolled < 2 patients per year (Tang C, Sherman S, Price M, et al. Clinical Trial Characteristics and Barriers to Participant Accrual: The MD Anderson Cancer Center Experience over 30 years, a Historical Foundation for Trial Improvement. Clin Cancer Res. 2017;23(6):1414-1421). Accrual rate for a study was calculated by dividing the total number of enrolled patients by the number of months between study enrollment start and study enrollment end (using a time between dates online calculator). A primary endpoint was considered positive if the predefined statistical assumptions for the endpoint were met in the study.

© 2021 Das S et al. *JAMA Network Open.*

eFigure 1. PRISMA Diagram Depicting How the Studies Included in the Analysis Were Chosen.

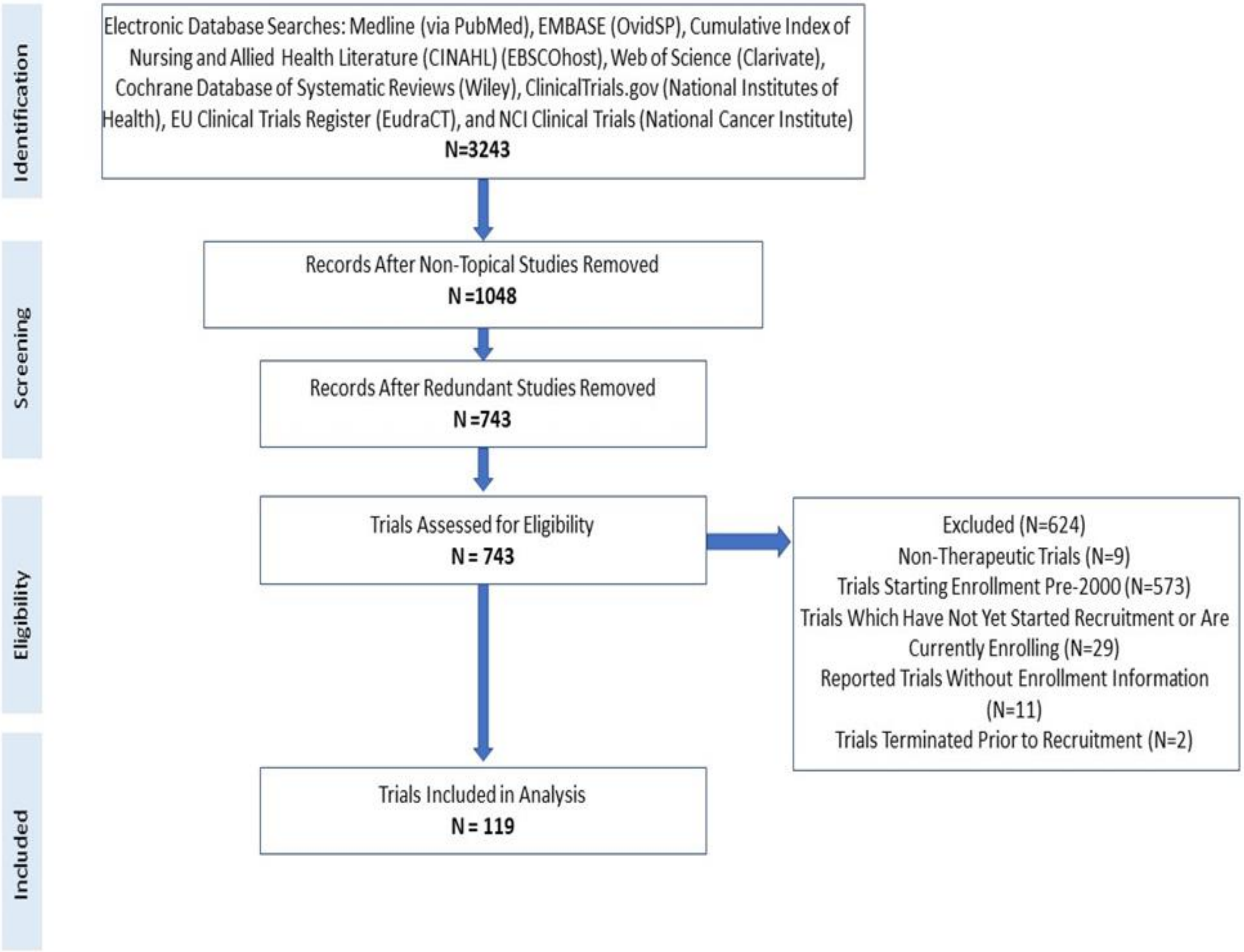

© 2021 Das S et al. *JAMA Network Open.*

eFigure 2. Comparison of Study Characteristics Between Currently Enrolling Trials and Completed Studies Included in Our Analysis

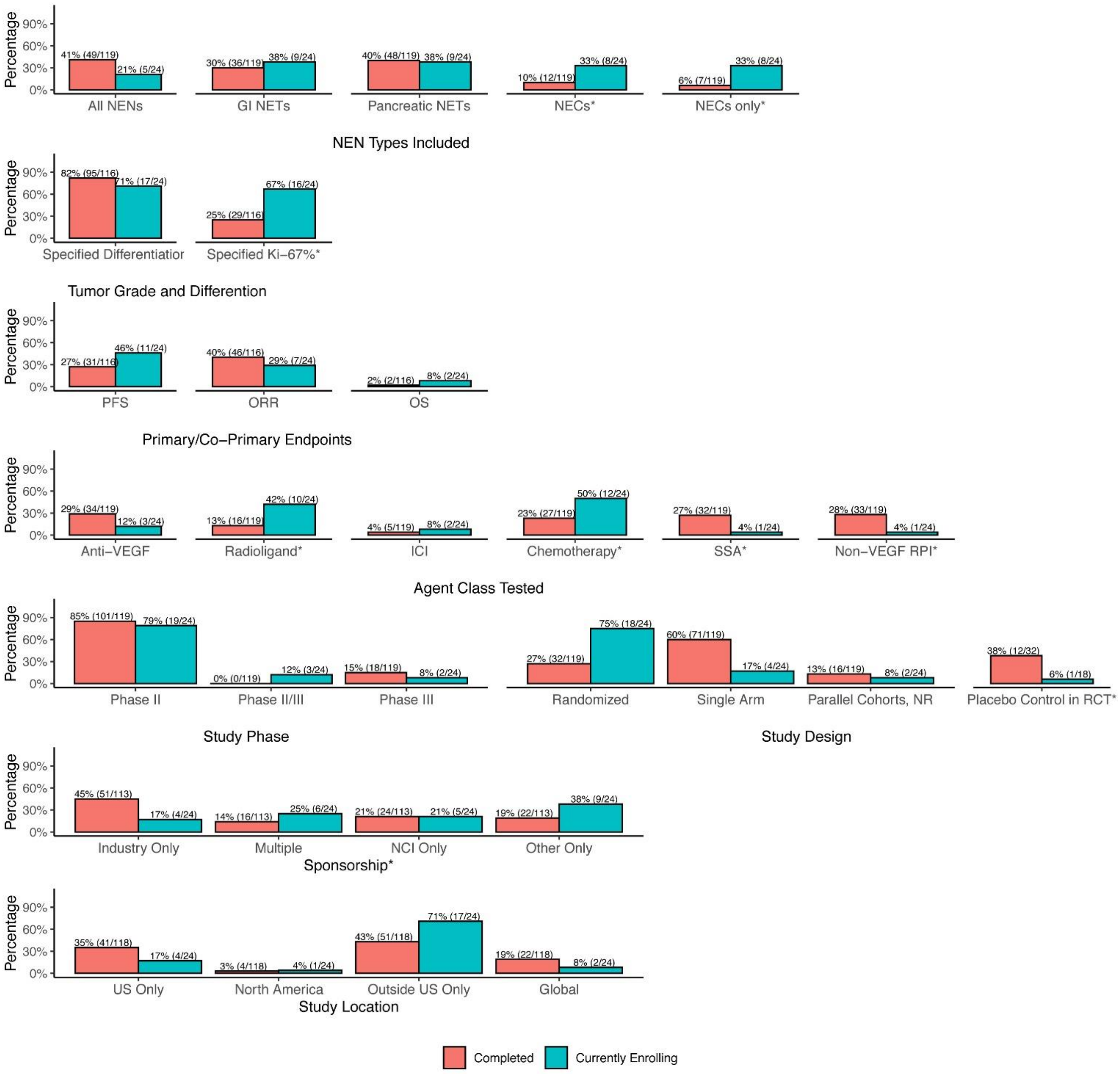

The asterix denotes statistically significant differences between the groups for a particular study characteristic.

© 2021 Das S et al. *JAMA Network Open.*

Abbreviations: ICI, immune checkpoint inhibitor, RPI, receptor pathway inhibitor; SSA, somatostatin analog; VEGF, vascular endothelial growth factor; NEN, neuroendocrine neoplasms; NETs, neuroendocrine tumors; NECs, neuroendocrine carcinomas; GI, gastrointestinal; RCT, randomized controlled trial; ORR, objective response rate; PFS, progression-free survival; OS, overall survival; NR, non-randomized.

© 2021 Das S et al. *JAMA Network Open.*
